# Measuring Chinese Middle School Students’ Motivation Using the Reduced Instructional Materials Motivation Survey (RIMMS): A Validation Study in the Adaptive Learning Setting

**DOI:** 10.3389/fpsyg.2020.01803

**Published:** 2020-08-04

**Authors:** Shuai Wang, Claire Christensen, Yuning Xu, Wei Cui, Richard Tong, Linda Shear

**Affiliations:** ^1^SRI International, Menlo Park, CA, United States; ^2^Kidaptive Inc., Redwood City, CA, United States; ^3^Squirrel AI Learning by Yixue Education Group, Shanghai, China

**Keywords:** motivation and engagement, survey, education technology, adaptive learning systems, factor analysis

## Abstract

Valid measures of student motivation can inform the design of learning environments to engage students and maximize learning gains. This study validates a measure of student motivation, the Reduced Instructional Materials Motivation Survey (RIMMS), with a sample of Chinese middle school students using an adaptive learning system in math. Participants were 429 students from 21 provinces in China. Their ages ranged from 14 to 17 years old, and most were in 9th grade. A confirmatory factor analysis (CFA) validated the RIMMS in this context by demonstrating that RIMMS responses retained the intended four-factor structure: attention, relevance, confidence, and satisfaction. To illustrate the utility of measuring student motivation, this study identifies factors of motivation that are strongest for specific student subgroups. Students who expected to attend elite high schools rated the adaptive learning system higher on all four RIMMS motivation factors compared to students who did not expect to attend elite high schools. Lower parental education levels were associated with higher ratings on three RIMMS factors. This study contributes to the field’s understanding of student motivation in adaptive learning settings.

## Introduction

Adaptive learning systems use various learning algorithms, such as machine learning and item response theories, to personalize the learning sequence for each student. Personalization is based on system-generated student learning profiles, which are informed by students’ performance on an initial knowledge diagnostic, and continuously updated with student usage data and learning behaviors. As students spend more time in the system, their learning profiles become more accurate and allow for greater personalization (Hauger and Köck, 2007, 355–60; Van Seters et al., 2012, 942–52). Well-known adaptive learning systems include Knewton, ALEKS, i-Ready, Achieve3000, and various cognitive tutor programs.

Studies show that adaptive learning systems are often effective at promoting student learning. A review found that 32 of 37 (86%) studies on the effects of adaptive learning reported positive learning achievement outcomes (Xie et al., 2019, 11). A comparison of 6,400 courses found that courses with adaptive assignments produce greater learning gains than courses without adaptive assignments (Bomash and Kish, 2015, 8–15). A large-scale effectiveness study found that an adaptive learning system improved the average student’s performance on an algebra proficiency exam by approximately 8 percentile points (Pane et al., 2014, 127). Another study found that over 2 years, personalized learning improved student mathematics performance, equivalent to a 3 percentile gain on a standardized assessment (Pane et al., 2017, 3).

The extent to which students benefit from adaptive learning systems may depend in part on their experiences of motivation while using such systems. Student motivation influences both the learning process and its outcomes (Pintrich and de Groot, 1990, 33–40; Zimmerman et al., 1992, 663–76; Carini et al., 2006, 1–32; Glanville and Wildhagen, 2007, 1019–41; Kuh et al., 2007, 1–5). Unfortunately, students’ motivation tends to decline as they progress through K-12 educational systems (Christidou, 2011). This decline has been linked to educational systems’ inadequate fulfillment of students’ needs for autonomy, self-efficacy, and relevance in their learning (Gnambs and Hanfstingl, 2016, 1698–1712). To prevent or reverse this trend, it is vital to understand where motivation is lost and for whom. Measuring student motivation may improve adaptive learning systems by enhancing the accuracy of student learning profiles, the basis for personalization.

One popular framework for measuring student motivation is the Attention Relevance Confidence Satisfaction (ARCS) model of student motivation. The ARCS model was developed in the context of face-to-face classroom instruction but has since been applied in computer- and Web-based instructional settings (e.g., Keller, 1999, 39–47; Astleitner and Hufnagl, 2003, 361–76). The ARCS Model posits that four factors must be present to motivate a student (Keller, 1987, 1–2):

• Attention: The learning experience must capture and hold the student’s attention, for example by stimulating curiosity and varying the presentation style.
• Relevance: The learning experience must feel personally relevant to the student. It might meet a specific need such as a credential, align with the student’s motives or values, or connect to familiar experiences.
• Confidence: The learning experience must elicit a sense of confidence in one’s ability to learn. It might do this by setting clear requirements for success and providing appropriately challenging opportunities.
• Satisfaction: The process or results of the learning experience must feel satisfying to the student. Learning experiences can promote satisfaction by providing meaningful opportunities to apply new skills, reinforcing students’ successes, and expressing that all students are evaluated using the same standards.

The ARCS model has implications both for measuring students’ motivation and for improving educational interventions to enhance motivation. Keller’s Motivational Tactics Checklist (Keller, 2010, 287–91) describes strategies for supporting each of the four factors of the ARCS Model. User instructions designed to support even one factor of the ARCS Model have been shown to improve performance over instructions that have not been manipulated to support motivation (Loorbach et al., 2007, 343–358).

The Instructional Materials Motivation Survey (IMMS) is the original measure of student motivation based on the ARCS model (Keller, 2010, 277–87). The IMMS is widely used to measure students’ needs prior to engaging with instructional materials, and to measure students’ reactions after engaging with instructional materials. It consists of 36 items arranged in four subscales corresponding to the ARCS model’s components of motivation. One validation study supported the validity of the IMMS, reporting Cronbach’s alpha ≥0.75 for all IMMS dimensions, interdimension correlations from 0.40 to 0.80, and statistically significant correlations with global satisfaction ratings (Cook et al., 2009, 1507–8). Meanwhile, three studies found that it is necessary to reduce the number of items on the IMMS to strengthen the measure’s psychometric properties (Huang et al., 2006, 250; Loorbach et al., 2015, 9; Hauze and Marshall, 2020, 49–64).

The 12-item Reduced IMMS (RIMMS) consists of three items measuring each of the four IMMS subscales, respectively. A validation study found that the RIMMS fits the four factors of the ARCS Model better than the IMMS (Loorbach et al., 2015, 204–218). The RIMMS is appropriate for measuring students’ responses to adaptive learning technology; it was validated in an individual learning setting (Loorbach et al., 2015, 204), and has been applied in a range of computer-based learning settings (Khacharem, 2017, 4; Linser and Kurtz, 2018, 1508; Nel and Nel, 2019, 178–80; Villena Taranilla et al., 2019, 6). These studies have generally reported acceptable subscale reliabilities for each of the four subscales: attention (α = 0.73–0.90), relevance (α = 0.69–0.82), confidence (α = 0.59–0.89), and satisfaction (α = 0.82–0.88). These studies have also found evidence of measure sensitivity: they have detected pre- intervention- to post-intervention change.

The RIMMS was validated in the Netherlands (Loorbach et al., 2015, 1) and has since been applied in Spain, the United States, Holland, the United Kingdom, Canada, and South Africa (Linser and Kurtz, 2018, 1508; Nel and Nel, 2019, 178; Villena Taranilla et al., 2019, 4). Yet no studies to date have investigated its psychometric properties in Eastern cultures. Validation is essential when applying a measure in a new culture, as culture influences both behavior and cognition (Han, 2010, 297–88; Varnum et al., 2010, 9–13; Millar et al., 2013, 138–57). Consequently, instruments do not necessarily retain their psychometric properties when used in new cultural contexts (Hambleton et al., 2005; Dai et al., 2020, 3–5). For example, researchers who translated the IMMS into Turkish found that it required substantial modification to exhibit sound psychometric properties (Kutu and Sozbilir, 2011, 292).

The present study aims to (1) validate the RIMMS (Loorbach et al., 2015, 204–18) in a sample of Chinese middle school students using an adaptive learning system, and (2) examine associations among student motivation and student background characteristics in this setting. While China’s online education market is large, with 144 million online education users in 2017 (China Internet Network Information Center, 2017), adaptive learning is relatively new to this market. Accordingly, research on students’ motivation while using adaptive learning systems in China is nascent. Validating a measure of student motivation for this context is key to improving adaptive learning systems to support Chinese students’ motivation and learning.

In addition to validating the RIMMS in this sample, the present study aims to illustrate the utility of student motivation measures for identifying sub-groups of students who may require motivational support. For example, patterns of motivation or engagement may differ by educational aspirations (Roebken, 2007, 3–9; Gutman and Schoon, 2018, 114–15), socioeconomic status (Willms, 2003, 48), gender (Hsieh et al., 2015, 341–45), or familiarity with the learning format (Miller et al., 2011, 1431; Orfanou et al., 2015, 238). Detecting these differences may improve the precision with which adaptive learning systems can target motivational support.

## Materials and Methods

### Participants

Participants were 429 students from 21 provinces in China. Recruitment targeted a wide range of provinces and schools. The study included all schools and students that agreed to participate. Students’ ages ranged from 14 to 17. Most students were in 9th grade. They represented typical students of their age in their provinces. As incentives, students received school supplies (e.g., pens, ruler, and eraser).

### Procedures

The study took place in 21 provinces. All participants went to designated schools or learning centers in their provinces and received free transportation and boarding if necessary. Over two consecutive days in the summer, participants used Squirrel AI Learning for a total of 6 h and 40 min. Participants followed identical learning schedules, including times for studying, breaks, and lunch. The schedule was designed to maximize time spent in the Squirrel AI Learning system. Participants used Squirrel AI Learning individually in school computer labs; they did not receive human instruction or tutoring. A researcher monitored each computer lab to provide technical assistance and to ensure that students followed the schedule. An independent research organization monitored study implementation by proctoring exams and enforcing uniform learning schedules.

### Measures

Before students used Squirrel AI Learning, they were provided with a basic-information questionnaire, which included student information (e.g., year of birth, gender, grade level) and family background information (e.g., parent education level).

After using Squirrel AI Learning, students were asked to complete a paper version of the RIMMS in Chinese. The RIMMS was administered after students used Squirrel AI Learning to measure students’ experiences of motivation while using the system, consistent with prior studies using the RIMMS and IMMS (Loorbach et al., 2015, 204, 211). The research team translated the English version of RIMMS into Chinese, and back translated to guarantee the accuracy of the survey (see Table 1). Students were given a maximum of half an hour to complete the survey, but most students completed the survey within 10 min.

**TABLE 1:** Standardized factor loadings based on CFA (*N* = 417).

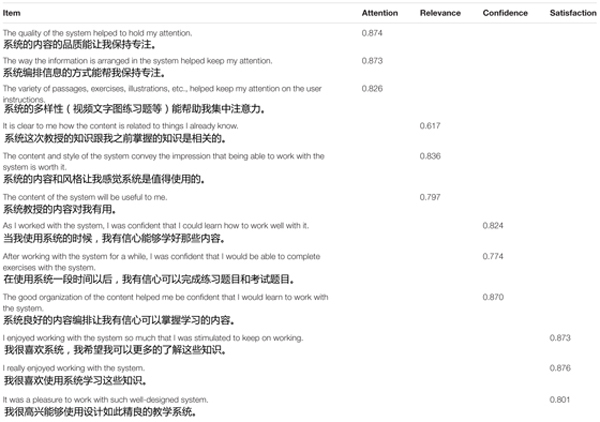

*Only the Chinese translation was presented to students.*

### Instructional Materials

One of the first Chinese developers to release an adaptive learning system, Squirrel AI Learning has established over 2,000 learning centers in over 700 cities serving almost 2 million registered accounts. Users represent a range of socioeconomic status, urbanicity, and academic achievement. For a more detailed description of the Squirrel AI Learning System, see Li et al. (2018), 46–8.

Participants studied Squirrel AI Learning 9th grade math content. The content was considered review for the students, consistent with the predominant use of Squirrel AI Learning in China. Topics included quadratic equations, parallelograms, and linear functions.

Squirrel AI Learning’s product design is grounded in many of the same design principles specified in the ARCS Model as motivation supports (Keller, 1987, 2–7). Of the ARCS Model’s four motivational factors, the Squirrel AI Learning system is primarily designed to support student confidence and attention.

The Squirrel AI Learning system’s adaptive learning technology presents each student with problems targeted to their ability, so that problems are neither too challenging nor too simple. This is consistent with Keller’s recommendations for supporting student confidence, based on the ARCS Model: “The success experience will be meaningful and will stimulate continued motivation if there is enough challenge to require a degree of effort to succeed, but not so much that it creates serious anxieties or threatens failure” (Keller, 1987, 4).

Keller (1987), 5 also specified that instructional materials can support confidence by providing corrective feedback. The Squirrel AI Learning system includes an intelligent, immediate feedback mechanism. After completing each problem, students are told whether they have answered correctly, with elaborated explanations and opportunities to correct their work. Research has demonstrated the benefits of such instant and frequent feedback (Hattie and Timperley, 2007, 81–112).

To support students’ attention, Keller recommends promoting an inquiry mindset by engaging students in problem solving (Keller, 1987, 2–3). The Squirrel AI Learning system aims to foster an inquiry mindset through its learn-by-doing approach, in which students learn by solving problems, with the option to use additional resources as needed. The Squirrel AI Learning system uses the student’s learning profile to select the most appropriate learning resources for the student, such as instructional videos, lecture notes, and worked examples. Using a variety of instructional supports further supports students’ attention (Keller, 1987, 2–3).

### Analysis

**FIGURE 1:**
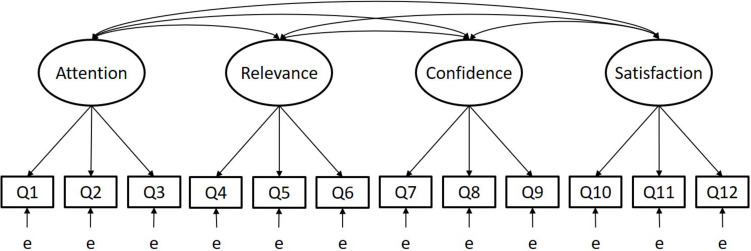
Factor and item relationships tested in confirmatory factor analysis.

To answer RQ1, we used confirmatory factor analysis (CFA), widely considered a rigorous quantitative approach to validate instruments (Wang and Wang, 2012). We specified the four-factor model proposed in RIMMS (Figure 1) and applied several criteria to determine whether the data fit this model. While 0.3 is a conventional threshold for factor loadings (Brown, 2006), to be conservative we only considered a factor acceptable if it met a higher factor loading threshold of 0.4 (Ford et al., 1986). In addition to examining the value of factor loadings, we also investigated whether each factor loading demonstrated statistical significance. Furthermore, we examined indices of model fit: Standardized Root Mean Square Residual (SRMSR) <0.05 indicates close approximate fit; Comparative Fit Index (CFI) of >0.9 represents reasonably good fit; Tucker Lewis Index (TLI) >0.9 indicates reasonably good fit (see Hu and Bentler, 1999). Finally, we examined Cronbach’s alpha to assess reliability of the factors, applying reliability threshold of >0.7, a widely used threshold in educational studies (Nunnally, 1978).

Next, for RQ2, we used a multiple indicators multiple causes (MIMIC) model, a type of structural equation model which relates CFA-based results to student and family background information. Based on our review of the literature, as well as consultations with expert teachers, the indicators selected for examination during the MIMIC modeling process included student gender, student familiarity with the content, student education aspiration, and parent education level. Specifically, gender was a binary variable (female or male; “male” was the reference level). Student familiarity with computers was a self-report binary variable (very familiar or not; “not familiar with computers” was the reference level). Student education aspiration was a binary variable (expect yourself to attend an elite high school or not; “not expecting to attend an elite high school” was the reference level). Parent education level was a binary variable (high = at least one parent’s highest degree was college or above, or low = neither parents attended college or above; low was the reference level). Student grade level was excluded from the analysis because 96% of the sample were grade 9 students and there was little variance in the variable.

The analysis team analyzed only a de-identified dataset. All analyses were completed in Mplus.

## Results

A total of 417 students completed the Chinese version of RIMMS. For RQ1, with an RMSEA of 0.059, the four-factor CFA demonstrated close fit with the data (Byrne, 1998). CFA analyses also indicated robust item loadings (all larger than 0.6) on this model for the validation data (see Table 1), and all factor loadings were significant (*p* < 0.001). In addition, indices of model fit were very robust: the final model showed an SRMSR of 0.024 (values <0.05 indicate close approximate fit), a CFI of 0.984 (values >0.9 represent reasonably good fit), and a TLI of 0.978 (values >0.9 indicate reasonably good fit). In addition, Cronbach’s Alpha indicated high reliabilities for the four factors of RIMMS: Cronbach’s alpha is 0.89 for attention, 0.80 for relevance, 0.86 for confidence, and 0.89 for satisfaction (Nunnally, 1978). This indicated that the four-factor RIMMS model is valid for making inferences about student motivation among Chinese 9th grade students in an adaptive learning setting.

**FIGURE 2:**
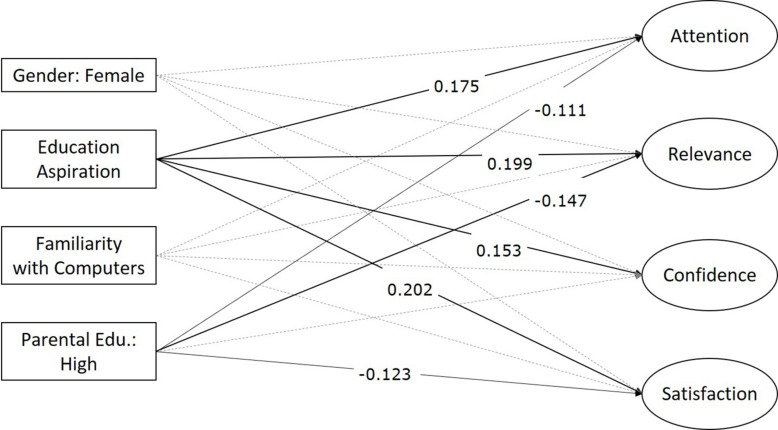
Associations between student background and RIMMS. Insignificant loadings were excluded from the figure.

For RQ2, a total of 397 students provided complete information on both the RIMSS and the background survey. The MIMIC model demonstrated close fit with the data: the final model showed a SRMSR of 0.026, a CFI of 0.978, and a TLI of 0.969. Based on the MIMIC model, educational aspiration was positively related to all four RIMMS factors: attention (*B* = 0.380, *p* < 0.01), relevance (*B* = 0.432, *p* < 0.01), confidence (*B* = 0.333, *p* < 0.01), and satisfaction (*B* = 0.148, *p* < 0.01). Parental education level was negatively related to attention (*B* = −0.235, *p* < 0.05), relevance (*B* = −0.311, *p* < 0.01), and satisfaction (*B* = −0.260, *p* < 0.05), but not confidence (*p* = 0.299). Neither student familiarity with computers nor student gender were significantly associated with the motivation factors. See Figure 2 for details.

## Discussion

Motivation is integral to academic success (Pintrich and de Groot, 1990, 33–40; Zimmerman et al., 1992, 663–76; Carini et al., 2006, 1–32; Glanville and Wildhagen, 2007, 1019–41; Kuh et al., 2007, 1–5). Measuring motivation is foundational to improving it. This study validated a measure of student motivation, the Reduced Instructional Materials Motivation Survey (RIMMS), in a sample of Chinese middle school students using an adaptive learning system for math content review. A CFA found evidence of validity: RIMMS responses retained the four-factor structure intended by the measure’s developers – attention, relevance, confidence, and satisfaction. This finding adds to the growing body of evidence suggesting the RIMMS can be used to draw valid inferences in a variety of cultures (Linser and Kurtz, 2018, 1508; Nel and Nel, 2019, 178; Villena Taranilla et al., 2019, 4). This is the first study, to our knowledge, that extends these findings to China. As adaptive learning systems become increasingly popular in China, such measures can be used to design systems that adapt not only to students’ learning progress, but also to their motivational needs.

This study illustrates how the RIMMS can be used to identify differences in students’ motivation profiles. Findings are consistent with prior research highlighting the relationship between self-efficacy and academic goal setting (Bandura and Schunk, 1981). Students with higher education aspirations (who expected to attend elite high schools) rated the Squirrel AI Learning system higher on all four RIMMS motivation factors: attention, relevance, confidence, and satisfaction. These students may have felt that Squirrel AI Learning math content was relevant to their educational aspirations. They may have had greater prior mathematical ability, resulting both in higher educational aspirations and confidence when using Squirrel AI Learning. They may also have foreseen or experienced satisfying opportunities to apply these skills in pursuit of admission to elite high schools.

Further, the RIMMS was able to detect differences in student motivation by parental education. Measuring socioeconomic differences in motivation is especially important given that students from families of low socioeconomic status are more likely to be disengaged in school, a pattern that has been reported in both Eastern and Western cultures (Willms, 2003, 48; Hannum and Park, 2004, 8–12). By contrast, the present study found that students who reported lower parental education rated the Squirrel AI Learning System higher on the attention, relevance, and satisfaction factors of motivation than participants who reported higher parental education. This novel finding illustrates the utility of measuring product- or situation-specific motivation to reveal nuances in broader trends.

In this study, gender did not predict students’ RIMMS ratings of an adaptive learning system. Given that gender stereotypes negatively affect girls’ math performance in both Eastern and Western cultures (Beilock et al., 2010, 1861; Song et al., 2016, 943–952), it is encouraging that male and female students reported similar motivational experiences using Squirrel AI Learning. More research is needed to clarify the conditions under which gender may affect students’ experiences with edtech. While some studies have found an association between gender and edtech engagement (Lowrie and Jorgensen, 2011, 2246–47; Hsieh et al., 2015, 341–45), others have found that males and females tend to perceive edtech similarly (Meiselwitz and Sadera, 2008, 238; Ituma, 2011, 61; Orfanou et al., 2015, 237). Validated measures of student motivation, such as the RIMMS, may be instrumental for identifying factors associated with gender differences in edtech engagement.

This study found that familiarity with computers did not predict students’ motivational experiences with an adaptive learning system. It may be that general computer familiarity is too low a bar to distinguish among 9th grade students. We included this variable because some participants were from rural areas with limited computer access, but most students reported they were familiar with computers. Other studies reporting associations between system familiarity and user experience have generally measured platform-specific experience, such as experience with educational games or learning management systems (Miller et al., 2011, 1428; Orfanou et al., 2015, 233).

Psychometrically sound measures of student motivation, such as the RIMMS, will enable researchers and developers to better understand students’ experiences with adaptive learning systems. This information could be used in efficacy studies to explore how outcomes vary by student engagement. It could also be useful for product development. In an adaptive learning system, motivation data could improve the accuracy of student learning profiles, facilitating a more personalized learning experience. For example, the system might learn that a student is low on a specific motivation factor, then boost that factor’s salience for that student by applying factor-specific strategies from Keller’s Motivational Tactics Checklist (Keller, 2010, 287–91). Adaptive learning systems will become increasingly powerful as they develop the capacity to personalize not only educational content but also motivational supports.

### Limitations

Some aspects of the study design limit the external generalizability of our findings. The current study focused only on 9th grade students and on a limited number of mathematics topics. Future studies should explore whether the RIMMS retains its four-factor structure, and whether associations between student characteristics and motivation hold, in other grades and subject areas. This study was conducted over a relatively short timeframe (2 days). Participants had not used the Squirrel AI Learning system before this study; it is possible that the study was too short for the novelty to wear off. The research team accepted these tradeoffs to arrange for a large sample of students from many regions of China to use the adaptive learning system at the same time. Finally, our findings regarding the association between parental education and motivation may not generalize to socioeconomic status more broadly. This study used student-reported parental education as a proxy for socioeconomic status because Chinese schools do not offer free or reduced-price lunch, and students may not be able to provide accurate information about parental income.

### Conclusion

Adaptive learning systems are often effective learning tools. While they are common in the United States and elsewhere, they are relatively new and gaining popularity in China. To develop effective, engaging systems, developers need information about students’ experiences. While validated measures of student motivation exist, little is known about their applicability in a Chinese adaptive learning context. This study found that the RIMMS can be used to draw valid inferences about Chinese 9th grade students’ experiences of motivation when using an adaptive learning system intensively over a short timeframe for math content review.

## Data Availability Statement

The datasets generated for this study are not publicly available due to restrictions set by our Institutional Review Board agreement. Participants did not consent to have their data shared.

## Ethics Statement

Written informed consent from the participants’ legal guardian/next of kin was not required to participate in this study in accordance with the national legislation and the institutional requirements. In addition, SRI International followed best ethical practice by analyzing de-identified data, and the SRI Institutional Review Board determined that SRI’s analysis of the de-identified data did not constitute human subjects research.

## Author Contributions

SW, CC, and YX contributed to conception and design of the study. SW and YX performed the statistical analysis. SW, CC, and LS wrote different sections of the manuscript and contributed to manuscript revision. All authors contributed to the article and approved the submitted version.

## Conflict of Interest

SRI International (authors: SW, CC, and LS) was contracted by Squirrel AI Learning by Yixue Education Group to conduct this work. YX was a data analyst at SRI International when the data were analyzed, and she is currently an employee at Kidaptive. WC and RT are employees of Squirrel AI Learning by Yixue Education Group.
